# mTOR Inhibitor Everolimus Modulates Tumor Growth in Small-Cell Carcinoma of the Ovary, Hypercalcemic Type and Augments the Drug Sensitivity of Cancer Cells to Cisplatin

**DOI:** 10.3390/biomedicines13010001

**Published:** 2024-12-24

**Authors:** Kewei Zheng, Yi Gao, Jing Xu, Mingyi Kang, Ranran Chai, Guanqin Jin, Yu Kang

**Affiliations:** 1 Obstetrics and Gynecology Hospital, Fudan University, Shanghai 200011, China; zhengkw983@163.com (K.Z.); gaoyi_sepnd@126.com (Y.G.); doctor_xj@163.com (J.X.); mykang14@fudan.edu.cn (M.K.); chairanran7664@fckyy.org.cn (R.C.); jinguanqin7889@fckyy.org.cn (G.J.); 2 Shanghai Key Laboratory of Female Reproductive Endocrine Related Diseases, Shanghai 200011, China

**Keywords:** small-cell carcinoma of the ovary, hypercalcemic type, targeted therapy, combination therapy, proliferation, apoptosis, autophagy

## Abstract

**Background**: Small-cell carcinoma of the ovary, hypercalcemic type (SCCOHT), is a rare and aggressive cancer with a poor prognosis and limited treatment options. Current chemotherapy regimens are predominantly platinum-based; however, the development of platinum resistance during treatment significantly worsens patient outcomes. Everolimus, an mTOR inhibitor, has been widely used in combination cancer therapies and has successfully enhanced the efficacy of platinum-based treatments. **Method**: In this study, we investigated the combined effects of everolimus and cisplatin on SCCOHT through both in vitro and in vivo experiments, complemented by RNA sequencing (RNA-seq) analyses to further elucidate the therapeutic impact. **Result**: Our findings revealed that everolimus significantly inhibits the proliferation of SCCOHT cells, induces cell cycle arrest, and accelerates apoptosis. When combined with cisplatin, everolimus notably enhances the therapeutic efficacy without increasing the toxicity typically associated with platinum-based drugs. RNA-seq analysis uncovered alterations in the expression of apoptosis-related genes, suggesting that the underlying mechanism involves autophagy regulation. **Conclusions**: Despite the current challenges in treating SCCOHT and the suboptimal efficacy of platinum-based therapies, the addition of everolimus significantly suppresses tumor growth. This indicates that everolimus enhances cisplatin efficacy by disrupting survival-promoting signaling cascades and inducing cell cycle arrest. Furthermore, it points to potential biomarkers for predicting therapeutic response.

## 1. Introduction

Small-cell carcinoma of the ovary, hypercalcemic type (SCCOHT) is a rare and aggressive malignancy that predominantly affects young women, with a reported incidence of approximately 1–2% of all ovarian cancers. The prognosis for patients diagnosed with SCCOHT is particularly poor, with a five-year survival rate often less than 30% [1,2]. This dismal outlook is compounded by the limited treatment options available, as traditional chemotherapy regimens have shown minimal efficacy [3,4]. Although various treatments have been proposed, there is no international consensus on drug therapy and monitoring. A multimodal approach including radical cytopenic surgery, platinum-based chemotherapy, whole abdominal radiotherapy, and high-dose chemotherapy with autologous stem cell salvage (HDC ASCT) is usually recommended [5,6]. Currently, treatment options for SCCOHT are very limited and patient survival is usually low. There is an urgent need to investigate new therapeutic agents to provide more therapeutic options and ultimately improve patient survival and quality of life.

The mechanistic understanding of the mammalian target of rapamycin (mTOR) signaling pathway has garnered significant attention due to its pivotal role in regulating cellular growth, proliferation, and survival [7,8,9]. mTOR is a serine/threonine kinase that integrates various environmental signals, including nutrient availability and growth factors, to modulate cellular processes [10]. The dysregulation of the mTOR pathway has been implicated in numerous malignancies, including ovarian cancer, where it contributes to tumorigenesis and progression [11,12]. Available findings also suggest that the PI3K/AKT/mTOR signaling pathway is also significantly enriched in SCCOHT [13]. Therefore, for SCCOHT, targeting this pathway for treatment may have unexpected effects that can occur.

Recent studies have highlighted the potential of mTOR inhibitors, such as everolimus, in enhancing the efficacy of conventional chemotherapeutics like cisplatin [14,15,16]. The combination of these agents has been shown to synergistically inhibit tumor cell proliferation and induce apoptosis, primarily through the suppression of the phosphoinositide 3-kinase (PI3K)/AKT/mTOR signaling axis [17,18,19]. However, the current literature reveals a gap in comprehensive evaluations of this combination therapy specifically in SCCOHT [5], underscoring the need for further investigation into the molecular mechanisms underlying their combined effects.

This study aims to investigate the therapeutic efficacy of the combination of everolimus and cisplatin in treating SCCOHT. Given the limited treatment options and poor prognosis associated with SCCOHT, there is an urgent need to explore novel therapeutic strategies. The primary objective of this research is to systematically evaluate the effects of this drug combination on cell proliferation, apoptosis, and the underlying mechanisms involved.

The significance of this research lies in its potential to fill a critical gap in the current understanding of SCCOHT treatment. By elucidating the synergistic effects of everolimus and cisplatin, this study aims to contribute valuable insights that could lead to improved therapeutic strategies for patients suffering from this aggressive cancer type. Ultimately, the findings may pave the way for more effective clinical interventions, enhancing patient outcomes and survival rates.

## 2. Materials and Methods

### 2.1. Reagents and Cell Line

Everolimus (S1102), cisplatin (S1166), and 3-MA (S2767) were purchased from Selleck Chemical (Houston, TX, USA). Antibodies against mTOR (66888-1-Ig, 1:5000), P62 (18420-1-AP, 1:5000), LC3 (14600-1-AP, 1:1000), and Beclin1 (11306-1-AP, 1:1000) were purchased from Proteintech (Wuhan, China). p-AKT (4060, 1:2000), AKT (9272, 1:1000), and p-mTOR (5536, 1:1000) were purchased from Cell Signaling Technology (Beverly, CA, USA). GAPDH (GB15002, 1:3000) and Ki67 (GB121141, 1:300) were purchased from Servicebio (Wuhan, China).

The SCCOHT-CH-1 [20] and COV434 [21] cells were obtained from the Shanghai Key Laboratory of Female Reproductive Endocrine Related Diseases, Obstetrics and Gynecology Hospital, Fudan University, Shanghai, China. SCCOHT-CH-1 cells were cultured in RPMI1640 medium (BasalMedia, Shanghai, China) with 20% fetal bovine serum (Excellbio, Suzhou, China) and 1% penicillin–streptomycin (NCM Biotech, Suzhou, China). COV434 cells were cultured in Dulbecco’s Modified Eagle Medium (DMEM) (BasalMedia, Shanghai, China) supplemented with 10% fetal bovine serum and 1% penicillin–streptomycin and then cultured in a humidified incubator at 37 °C with 5% CO_2_.

### 2.2. Cell Counting Kit 8 (CCK-8) Assay

To detect the cytotoxicity of everolimus and cisplatin, SCCOHT-CH-1 and COV434 cells were inoculated into 96-well plates at 6 × 10^3^ cells per well. Everolimus and cisplatin were diluted to gradient concentrations using complete media. The cells were cultured for 48 h. Cell viability was assessed using the Cell Counting Kit-8 assay Kit (NCM Biotech, Suzhou, China). The optical density (OD) of each well was measured at 450 nm.

### 2.3. Synergy Determination with SynergyFinder

SCCOHT-CH-1 and COV434 cells were inoculated into 96-well plates at a density of 6 × 10^3^ per well and treated as follows. Single inhibitors (everolimus, cisplatin) or combination inhibitors (everolimus and cisplatin) were analyzed according to the dose specified in the cytotoxicity test. The two drugs were diluted at different ratios: for SCCOHT-CH-1 cells, everolimus was diluted at 0, 10, 20, 30, 40, 50, and 60 μM and cisplatin was diluted at 0, 0.12, 0.5, 2, 8, and 32 μM; for COV434 cells, everolimus was diluted at 0, 15, 25, 35, 45, 55, and 65 μM and cisplatin was diluted at 0, 1, 3, 9, 27, and 81 μM. Cell viability was measured 48 h after drug administration. Drug synergy scores were calculated using the Inhibition Index by response surface model and zero interaction titer (ZIP) calculation method using online SynergyFinder 3.0 (https://synergyfinder.fimm.fi, accessed on 18 February 2024). ZIP synergy score greater than 10 points is considered to have synergy. The drug combination response heat map was also developed to further evaluate the drug combination benefit.

### 2.4. Colony-Forming Assays

The cells were inoculated in 6-well plates at a density of 4 × 10^3^ per well. After 48 h of drug treatment, the cells were incubated in complete culture medium for 7–14 days. The cells were then washed with Phosphate-Buffered Saline (PBS) (BasalMedia, Shanghai, China) 3 times and fixed with 4% paraformaldehyde (Servicebio, Wuhan, China) solution for 30 min. Finally, dyed the cell with 0.1% crystal violet solution (Servicebio, Wuhan, China) for 30 min. The cells were observed under a microscope and quantified using ImageJ (version 2.14.0). Colony formation rate = the number of colonies formed after treatment/the number of colonies formed in control group × 100%.

### 2.5. Wound Healing Test

To examine the migration ability of the cells, a wound healing assay was performed. A total of 1 × 10^7^ cells per well were inoculated into the 12-well plate. After the cells were attached to the wall, each well was scratched with a pipette suction and replaced with a pure medium without fetal bovine serum. At 0 and 48 h after different treatments, the wounds were photographed and the open wound area was measured by ImageJ (version 2.14.0).

### 2.6. Cell Apoptosis Experiment

To detect the apoptosis rate of cells after different treatments, flow cytometry was performed using the apoptosis kit (Liankebio, Hangzhou, China). After different treatments, cells were collected and re-suspended in a 500 μL 1× Binding Buffer. Then, a stained incubation solution containing 5 μL of Annexin V-APC and 10 μL of Propidium iodide (PI) was prepared and incubated with the rehung cells in the dark for 5 min. The cell samples were analyzed by flow cytometry and the results were analyzed by FlowJo (version 10.8.1).

### 2.7. Cell Cycle Detection

A cell cycle detection kit (Liankebio, Hangzhou, China) was utilized in our research to detect the cell cycle distribution according to the manufacturer’s protocol. The cultured cells were analyzed by a flow cytometer (Beckman Coulter, Brea, CA, USA), and the results were analyzed by FlowJo (version 10.8.1).

### 2.8. EdU Staining Assay

Cell proliferation was analyzed using EdU staining. The SCCOHT-CH-1 and COV434 cell lines were treated with different drugs for 48 h and tested using an EdU test kit (Beyotime, Shanghai, China) according to the methods provided by the manufacturer. The results were observed under fluorescence microscopy and the EdU-positive cells in different treatment groups were analyzed.

### 2.9. TUNEL Assay

A commercial kit (Servicebio, Wuhan, China) was used for the TUNEL assay. The paraffin sections were dripped with protease K (without DNase) at 20 μg/mL and incubated at 37 °C for 30 min. After washing with PBS for 3 times, the slices were incubated in 1× equilibrium buffer for 30 min and then incubated at 37 °C for 1 h with a mixture containing 50 μL biotin-DUTP-labeled solution and 3 μL TdT enzyme. The slides were then incubated with 100 μL termination buffer for 10 min and then rinsed with PBS 3 times. The specimen was covered with streptavidin-HRP for 30 min, washed with PBS 3 times, and finally observed by microscope (BX63, Olympus, Tokyo, Japan).

### 2.10. Western Blotting Assay

When the cell density reached 80–90%, the cells were rinsed with pre-cooled PBS, and proteins were extracted using RIPA buffer containing protease and phosphatase inhibitors (Beyotime, Shanghai, China) and quantified using a BCA protein assay kit (Beyotime, Shanghai, China). Protein samples were boiled at 100 °C for 15 min. Each protein sample was separated by 10% SDS-PAGE gel at 30 μg and transferred to nitrocellulose membrane. After sealing with TBST of 5% skim milk for 1 h, the membrane was incubated with the corresponding primary antibody at 4 °C overnight. After TBST washing 3 times, it was incubated with secondary antibody at room temperature for 1 h. Detection was performed by enhanced chemiluminescence (NCM Biotech, Suzhou, China), and quantitative analysis was performed using ImageJ (version 2.14.0).

### 2.11. In Vivo Xenograft Model

The animal study protocol was approved by the Institutional Review Board of Department of Laboratory Animal Science, Fudan University (protocol code: 202308006S). Five-week-old female nude mice (BALB/c-nu) (Sipeifu, Beijing, China) were all reared without specific pathogen (SPF). 1 × 10^6^ SCCOHT-CH-1 cells were injected subcutaneously into mice. When the tumor volume reached 100 mm^3^, the mice were randomly divided into 4 groups of 6 mice each (PBS, everolimus, cisplatin, and combined therapy of everolimus and cisplatin). Everolimus (dissolved in DMSO/PEG300/Tween80/ddH_2_O, 0.5/30/5/64.5, *v*/*v*/*v*/*v*) was given intragastrically at 5 mg/kg twice a week; cisplatin (dissolved in ddH_2_O) was administered intraperitoneally at 3 mg/kg twice weekly. The weight and volume of the tumor were assessed [formula: tumor volume (mm^3^) = (length) × (width)^2^ × 0.5], and the weight of the mice was assessed. After euthanizing the mice, we collected the major organs for further toxicity analysis.

### 2.12. Immunohistochemical Staining (IHC) and Hematoxylin–Eosin Staining (HE)

When the tumor volume of mice reached 1500 mm^3^, euthanasia was performed, tumor tissues were collected, and vital organs were dissected for drug toxicity analysis. These specimens were fixed for 24 h using 4% paraformaldehyde (PFA). Subsequently, the fixed tissue was embedded in paraffin wax. Hematoxylin–eosin (HE) staining was performed on animal tumors and visceral tissues according to manufacturer’s instructions (G1005, Servicebio, Wuhan, China). Deparaffin and rehydrated sections of tumor tissue were heated in citrate buffer at 121 °C for 30 min to restore antigenic activity. The slices were incubated with a methanol solution of 0.3% hydrogen peroxide for 30 min to inhibit endogenous peroxidase activity. After blocking the non-specific reaction with 10% normal bovine serum, sections were incubated with KI67-specific rabbit polyclonal antibodies at 4 °C for 12 h. Then, the sections were washed with PBS and the secondary antibodies conjugated with horseradish peroxidase were incubated at 37 °C for 2 h. The stained sections were imaged under an inverted phase contrast microscope (BX63, Olympus, Japan).

### 2.13. Evaluation of Autophagy Flux

SCCOHT-CH-1 and COV434 cells were inoculated in 24-well plates. After the cell density reached 50–60%, the supernatant was discarded and rinsed once with Hank’s balanced salt solution (HBSS) (BasalMedia, Shanghai, China). DAPGreen (D676, Dojindo, Tokyo, Japan) Working Solution was added to each well and the cells were cultured at 37 °C for 30 min. Discard the supernatant and rinse twice with HBSS. The cells were treated with indicated drugs, cultured at 37 °C for 24 h, rinsed with HBSS twice, stained with Hoechst (C1017, Beyotime, Shanghai, China) for 10 min, rinsed with HBSS twice, and observed under fluorescence microscope (IX73, Olympus, Tokyo, Japan).

### 2.14. RNA Sequencing and Differentially Expressed Genes Analysis

The libraries were sequenced on an Illumina Novaseq 6000 platform and 150 bp paired-end reads were generated. About 51.16 M raw reads for each sample were generated. Raw reads of fastq format were first processed using fastp [22] and the low-quality reads were removed to obtain the clean reads. Then, about 46.89 M clean reads for each sample were retained for subsequent analyses. The clean reads were mapped to the human reference genome using HISAT2 [23]. FPKM [24] of each gene was calculated and the read counts of each gene were obtained by HTSeq-count [25].

Differential expression analysis was performed using the DESeq2 [26]. *p* value < 0.05 and log2(FC) > 2 or log2(FC) < −2 was set as the threshold for significantly differential expression genes (DEGs). Hierarchical cluster analysis of DEGs was performed using R (version 4.2.1) to demonstrate the expression pattern of genes in different groups and samples.

Based on the hypergeometric distribution, GO [27] and KEGG [28] pathway enrichment analysis of DEGs were performed to screen the significant enriched term using R (version 4.2.1), respectively. R (version 4.2.1) was used to draw the lollipop chart of the significant enrichment term.

Gene Set Enrichment Analysis (GSEA) was performed using GSEA software (version 4.3.3) [29,30]. The analysis was conducted using a predefined gene set, and the genes were ranked according to the degree of differential expression in the two types of samples. Then, it was tested whether the predefined gene set was enriched at the top or bottom of the ranking list.

### 2.15. Statistical Analysis

All statistical analyses were performed using Prism 10.1.0 (GraphPad, San Diego, CA, USA). R software (version 4.2.1) was utilized to carry out relevant statistical analyses in this study. All experiments and analyses were performed in triplicate, and the results are presented as mean ± standard deviation (SD). Unpaired Student’s *t*-test was used to compare two groups, and one-way ANOVA was used for comparisons between three or more groups. Statistical significance was * *p* < 0.05, ** *p* < 0.01, and *** *p* < 0.001.

## 3. Results

### 3.1. Everolimus Synergistically Enhanced the Cytotoxicity of Cisplatin Against SCCOHT Cells

**Figure 1:**
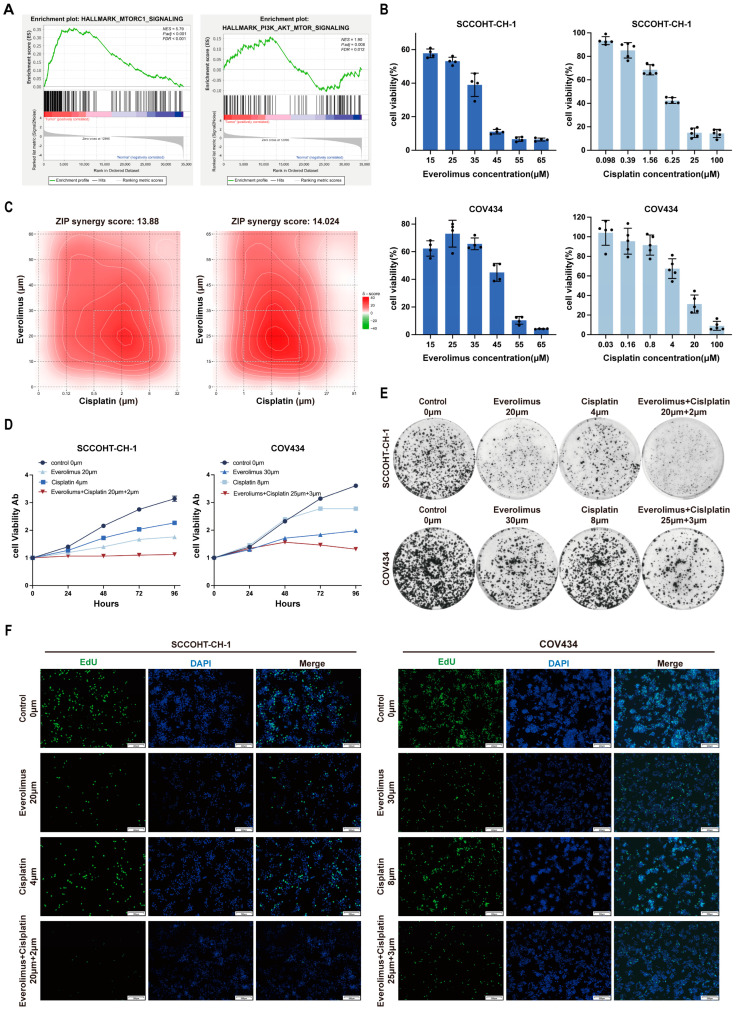
Effect of everolimus combined with cisplatin on proliferation of SCCOHT cells. (**A**) GSEA enrichment analysis. (**B**) IC50 of everolimus and cisplatin against SCCOHT cells. Data are expressed as mean ± SD. (**C**) Heatmaps of drug combination responses. Everolimus and cisplatin act synergistically in SCCOHT-CH-1 cells. Everolimus and cisplatin at the indicated concentrations were used to treat cells for 48 h, and cell viability was assessed by CCK-8 assay. The ZIP synergy scores were calculated using SynergyFinder 3.0, with scores greater than 0 indicating synergism and scores exceeding 10 reflecting a strong synergistic interaction. The white rectangle on the heatmap delineates the concentrations that correspond to the highest degree of synergy. (**D**) The proliferation of everolimus and cisplatin on SCCOHT cells was assessed by CCK-8 assay. (**E**) The colony forming ability of SCCOHT cells treated with everolimus or cisplatin was assessed. (**F**) EdU staining was used to analyze the effects of everolimus and cisplatin on the proliferation of SCCOHT cells (Scale bar = 200 μm).

Consistent with the previous literature [13], the RNA-seq sequencing data from our SCCOHT cohort also demonstrated that the PI3K/AKT signaling pathway is enriched in SCCOHT patients (Figure 1A). We evaluated the effects of everolimus and cisplatin on SCCOHT cell lines (SCCOHT-CH-1 and COV434). First, we assessed the cell viability of SCCOHT cells treated with different concentrations of everolimus using the CCK-8 method and plotted the dose–response curve (Figure 1B). The results showed that both everolimus and cisplatin inhibited the proliferation of SCCOHT cells in a dose-dependent manner. The half-maximal inhibitory concentrations (IC50s) of everolimus for SCCOHT-CH-1 and COV434 were 20.45 ± 0.271 μM and 33.19 ± 0.436 μM, respectively, while the IC50s for cisplatin were 4.32 ± 0.191 μM and 8.76 ± 0.305 μM, respectively. In the following experiments, we assessed the ability of everolimus to potentiate the cytotoxic effects of cisplatin on SCCOHT cells. The results from in vitro assays indicated that both everolimus and cisplatin displayed anti-proliferative effects that were dependent on their concentrations. We employed SynergyFinder software to calculate the ZIP synergy scores derived from the concentration gradients and the corresponding inhibition rates of the drugs (Figure 1C). The analysis demonstrated that the average anti-tumor response resulting from the drug combinations was 13.88 for SCCOHT-CH-1 cells and 14.024 for COV434 cells, suggesting a substantial synergistic effect on tumor growth suppression (ZIP synergy score > 10). As shown in Figure 1C, the white rectangular area delineates the region of optimal synergy. Specifically, SCCOHT-CH-1 cells were treated with everolimus at 20 μM in combination with cisplatin at 2 μM, while COV434 cells were treated with everolimus at 25 μM and cisplatin at 3 μM in subsequent in vitro studies. We further confirmed the synergistic effect using Combenefit software (version 2.021), which yielded analogous results (Figure S1A). Additionally, we assessed the effects of different treatment regimens on cell viability (Figure 1D) and colony formation ability (Figure 1E and Figure S1B). The CCK-8 results indicated that after 96 h, the combination of everolimus and cisplatin showed a significantly better anti-proliferative effect than either treatment alone. Similar results were obtained through colony formation assays and EdU experiments (Figure 1F, Figures S2 and S3).

### 3.2. Everolimus Co-Stimulation with Cisplatin Induced Apoptosis and Cell Cycle Arrest in SCCOHT Cells

**Figure 2:**
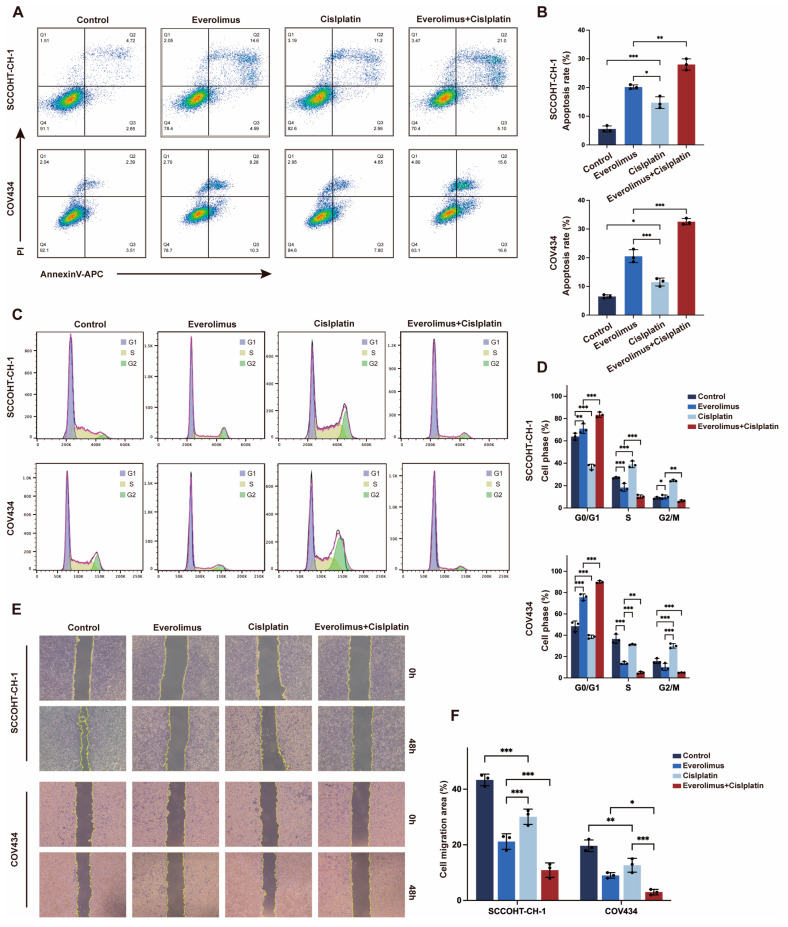
Everolimus in combination with cisplatin induced cell cycle arrest and increased apoptosis in SCCOHT cells. (**A**,**B**) apoptosis, (**C**,**D**) cell cycle analysis, and (**E**,**F**) wound healing assay. Error bars represent the standard deviation (Scale bar = 100 μm). Error bars represent the standard deviation, * *p* < 0.05, ** *p* < 0.01, *** *p* < 0.001.

To further explore the potential mechanisms of combination therapy, we analyzed the effects on cell apoptosis and the cell cycle using flow cytometry. The results showed that after the combination of everolimus and cisplatin, the apoptosis rate of SCCOHT cells significantly increased, particularly the proportion of late apoptotic cells (Figure 2A,B). Cell cycle analysis showed that the proportion of G0/G1 phase cells increased significantly after combination therapy (Figure 2C,D), suggesting that the combination of everolimus and cisplatin can effectively induce cell cycle arrest. Subsequently, we assessed the effect of everolimus on the migration ability of SCCOHT cells through a wound healing assay, and the results showed that the combination treatment significantly reduced the migration ability of the cells (Figure 2E,F).

### 3.3. The Combination of Everolimus Plus Cisplatin Enhanced the Inhibition of Tumor Growth in SCCOHT-CH-1 Xenograft Mouse Models

**Figure 3:**
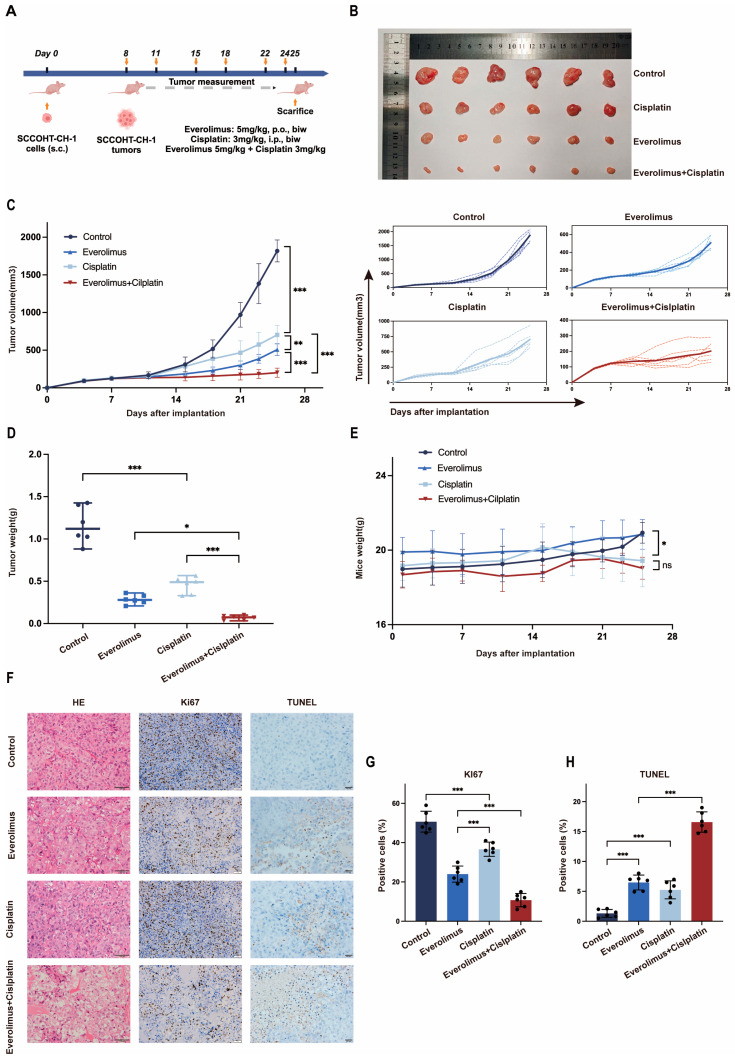
Everolimus combined with cisplatin enhanced the inhibition of tumor growth in vivo. (**A**) Administration regimen for tumor-bearing mice. (**B**) Mouse tumor images. Tumor volume and spaghetti curves of tumor volume (**C**), tumor weight (**D**), and mouse weight (**E**) in different groups of mice. (**F**–**H**) HE staining of tumor tissues, immunohistochemical analysis of Ki67 expression, and TUNEL staining in different groups of tumor tissues (Scale bar^HE, Ki67^ = 1000 µm, Scale bar^TUNEL^ = 500 µm). Error bars represent the standard deviation, ns: not significant, * *p* < 0.05, ** *p* < 0.01, *** *p* < 0.001.

To study the in vivo synergistic effect of everolimus and cisplatin, we used a subcutaneous tumor model in nude mice for subsequent experiments. As shown in Figure 3A, starting from the 8th day after SCCOHT-CH-1 cells were implanted into the nude mice, the mice were randomly divided into a control group, a cisplatin group (3 mg/kg), an everolimus group (5 mg/kg), and an everolimus + cisplatin group (5 mg/kg + 3 mg/kg), with treatment continuing for 18 days. Subsequently, the mice were euthanized, and tumor images are shown in Figure 3B (The detailed tumor volume data can be found in Table S1). During the treatment period, we continuously monitored the tumor volume (see Figure 3C) and mouse body weight (see Figure 3E). The results showed that the tumor volume in all four groups of mice gradually increased, but the tumor volume in the combination treatment group was significantly smaller than that in the other three treatment groups (control group, cisplatin group, and everolimus group). The tumor weight measurement results also showed a similar trend (see Figure 3D). Notably, everolimus treatment did not lead to weight loss in the mice, while all mice receiving cisplatin treatment experienced weight loss, and the combined use of everolimus did not further exacerbate the weight loss in the mice. The H&E staining results of tumor tissues from each group are shown in Figure 3F. Compared to the control group, the Ki67 positive rate in the treatment groups was significantly reduced, with the combination treatment group showing the most significant inhibitory effect. Similarly, TUNEL staining results indicated that the apoptosis rate of tumor cells in the everolimus + cisplatin group was the highest (see Figure 3F–H).

### 3.4. Toxicity Analysis

**Figure 4:**
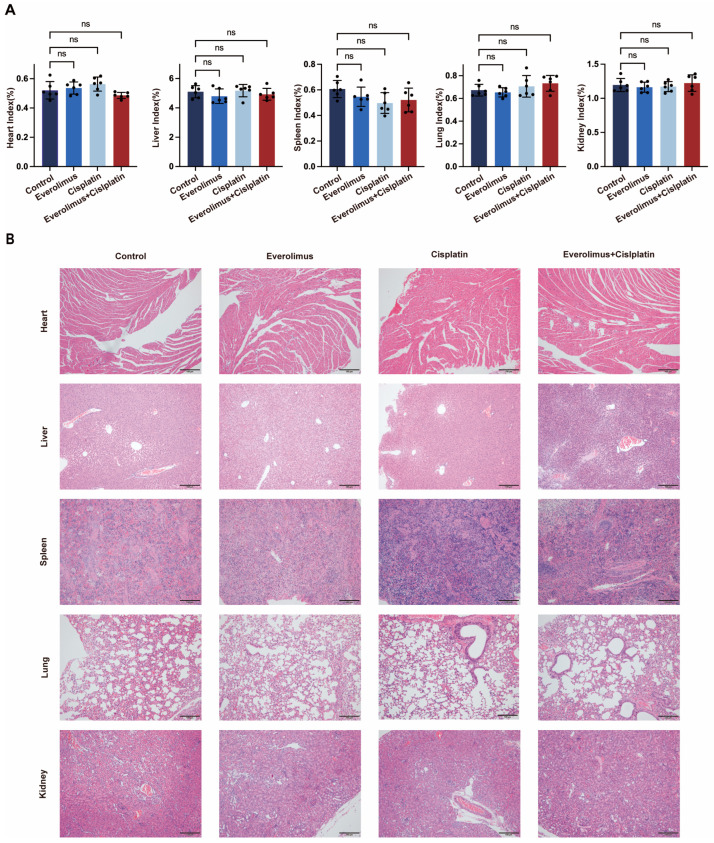
In vivo toxicity analysis. (**A**) The heart, liver, spleen, lung, kidney, and other organ indices of the animal at the end of the in vivo experiment. (**B**) HE staining of mouse organs (Scale bar = 100 µm). Error bars represent the standard deviation, ns: not significant.

By assessing the degree of damage to the major organs, we analyzed the systemic toxicity in four different groups. First, the major organs of the mice were dissected, weighed, and subjected to H&E staining to evaluate potential adverse reactions. As shown in Figure 4A, we calculated the organ index using the following formula: Organ Index (%) = [(Organ Weight)/(Mouse Body Weight)] × 100%. As shown in Figure 4B, histological analysis under a microscope was used to detect the toxicity of the drugs on important organs and their sections. Our results indicate that the major organs (such as the heart, liver, spleen, lung, kidney) did not show systemic toxicity, further validating the safety and reliability of our treatment regimen.

### 3.5. The Effect of Everolimus Is Mediated by the Autophagy

**Figure 5:**
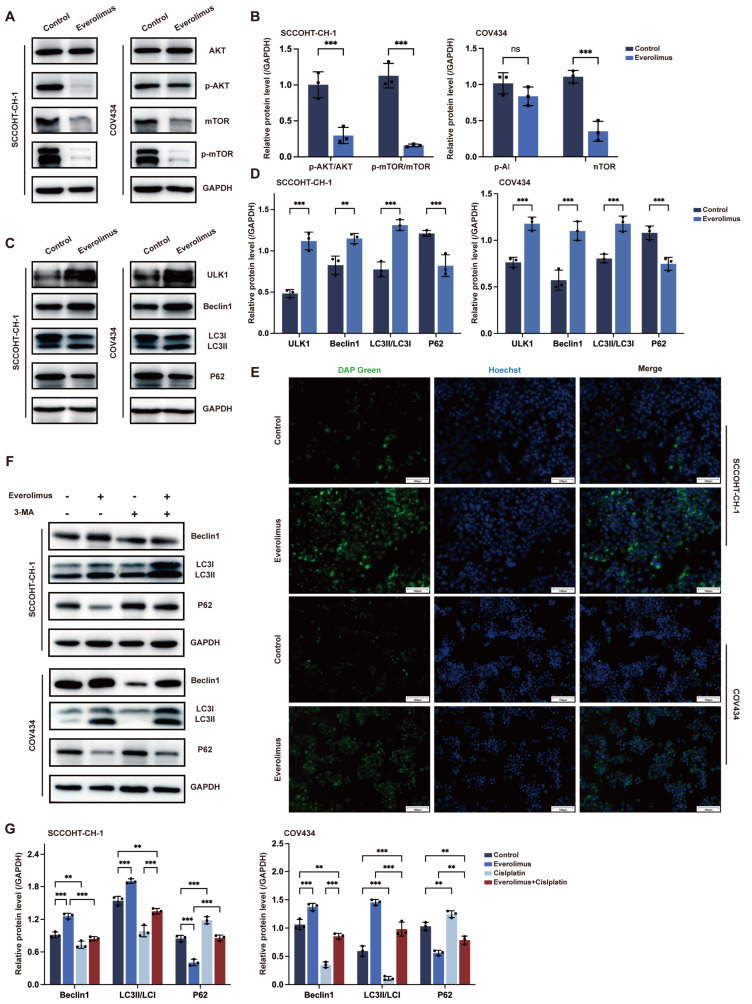
(**A**,**B**) Western blot analysis of phosphorylation of AKT and mTOR in SCCOHT cells of the control group and everolimus group. (**C**,**D**) Western blot analysis of changes in autophagy-associated protein levels in the control group and the everolimus group. (**E**) Representative images for the detection of the autophagy flux by staining with DAL^®^Green (green) (Scale bar = 100 µm). (**F**,**G**) The expression levels of autophagy related proteins and autophagy substrates in SCCOHT cells treated with everolimus, 3-MA, or everolimus + 3-MA were detected by Western blot analysis. Error bars represent the standard deviation, ns: not significant, ** *p* < 0.01, *** *p* < 0.001.

Next, we explored the mechanism by which everolimus inhibits the malignant behavior of SCCOHT. As an mTOR inhibitor, we first used Western blotting to detect the expression levels of mTOR and p-mTOR proteins. We found that everolimus simultaneously reduced the expression levels of both mTOR and p-mTOR proteins. As an upstream protein, AKT was also found to have its p-AKT protein expression level reduced by everolimus (Figure 5A,B). mTOR, as a key signaling molecule, plays an important role in cell growth and metabolism by regulating the initiation and execution of autophagy. Therefore, we further validated autophagy-related proteins. We treated SCCOHT-CH-1 cells with WX390 at different dosages and times, and found that autophagy levels were highest when the drug was treated at IC50 concentration for 24 h (Figure S5). The results of Western blotting showed that after treatment with everolimus, the expression of autophagy-related proteins ULK1, Beclin1, and LC3II/LC3I increased in SCCOHT-CH-1 and COV434 cells, while the expression level of p62 protein decreased (Figure 5C,D). The use of fluorescent small molecules to differentially stain autophagosomes and lysosomes further confirmed the results of the Western blot analysis (Figure 5E). To further verify the role of everolimus on autophagy activation in SCCOHT cells, we used the autophagy inhibitor 3-MA at a concentration of 10 µM to pre-treat the cells for 6 h. The results showed that in SCCOHT cells treated with 3-MA, the autophagy-related protein Beclin1 was significantly downregulated, and the conversion of LC3-II and the degradation of p62 were significantly inhibited. However, when cells were treated with both 3-MA and everolimus, the conversion of LC3-II and the degradation of p62 were partially restored compared to cells treated with 3-MA alone (Figure 5F,G), and the results of EdU staining and flow cytometry exhibited 3-MA rescued the apoptosis and proliferation inhibition induced by everolimus, indicating that everolimus-induced autophagy is cytodestructive autophagy (Figure S4).

### 3.6. Regulation of Apoptosis-Related Genes by Everolimus Combined with Cisplatin

**Figure 6:**
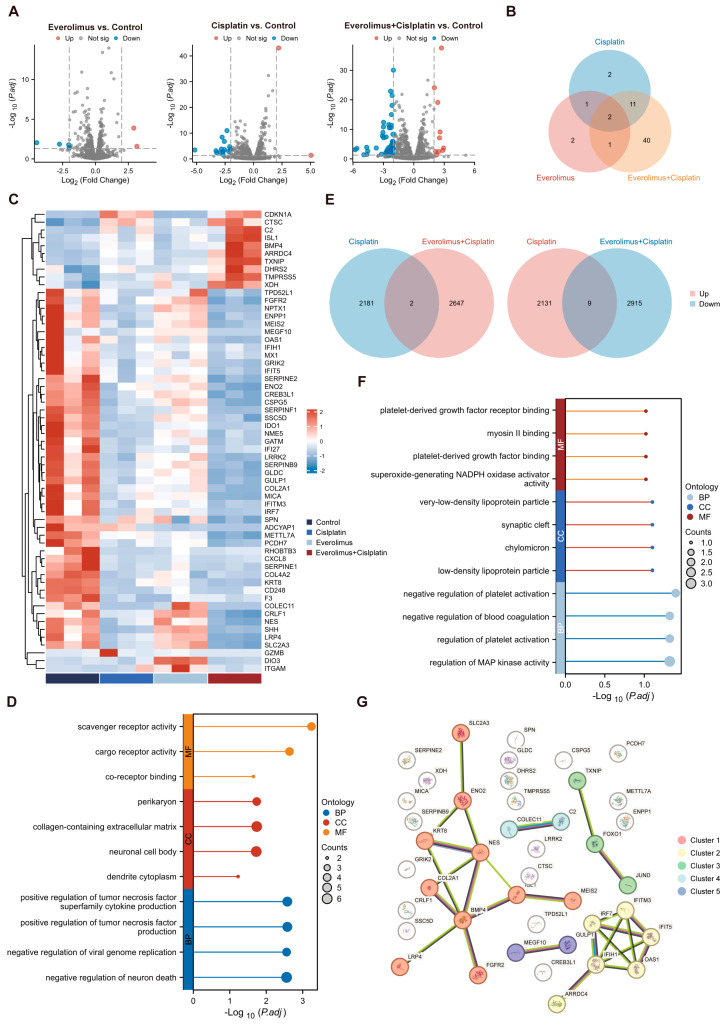
Regulation of apoptosis-related genes by everolimus and cisplatin. (**A**) Volcano maps showed differential expressions of apoptosis-related genes after different treatments. Genes with log2(FC) > 2 or log2(FC) < −2 are considered biologically significant. The red picture shows significantly upregulated gene (adjP < 0.05); The blue chart shows well downregulated genes (adjP < 0.05). (**B**) Venn diagram showed the differential expression of apoptosis-related genes after different treatments. (**C**) Heat maps showing the fold changes (logarithmic transformation) in apoptosis-related genes in different treatment groups. Calculate the fold change relative to the average of the control group. The shaded range from blue to red indicates downregulated genes to upregulated genes. (**D**) GO analysis of the 40 unique apoptosis-related genes. (**E**) Venn maps of upregulated and downregulated genes in cisplatin group and two-drug combination group. (**F**) GO analysis of the 11 reversed genes. (**G**) The PPI network for the 40 unique apoptosis-related genes and two specific reversed resistance genes.

To better understand the potential molecular mechanisms of everolimus combined with platinum-based therapy, we conducted RNA-seq analysis on xenograft model tumor samples and further analyzed autophagy-related genes (Figure S6) and apoptosis-related genes. Figure 6A shows the expression of apoptosis-related genes after different treatments, with six DEGs (two upregulated and four downregulated) after everolimus treatment, sixteen DEGs (two upregulated and fourteen downregulated) after cisplatin treatment, and a total of fifty-four genes showing significant differences in expression (ten upregulated and forty-four downregulated) after everolimus combined with cisplatin treatment. Human apoptosis-related genes were downloaded from GSEA (https://www.gsea-msigdb.org/gsea/index.jsp, accessed on 12 April 2024) and these gene sets are displayed in Table S2. The heatmap displays the fold changes in related genes to determine the overall transcriptomic differences in apoptosis-related genes (Figure 6C). There were significant changes in apoptosis-related genes after the combination of everolimus and cisplatin. Compared to the use of everolimus and cisplatin alone, the combination resulted in forty unique apoptosis-related genes, with nine genes upregulated (*ARRDC4*, *BMP4*, *C2*, *CTSC*, *DHRS2*, *ISL1*, *TMPRSS5*, *TXNIP*, *XDH*) and thirty-one genes downregulated (*COL2A1*, *COLEC11*, *CREB3L1*, *CRLF1*, *CSPG5*, *ENO2*, *ENPP1*, *FGFR2*, *GLDC*, *GRIK2*, *GULP1*, *IFIH1*, *IFIT5*, *IFITM3*, *IRF7*, *KRT8*, *LRP4*, *LRRK2*, *MEGF10*, *MEIS2*, *METTL7A*, *MICA*, *NES*, *OAS1*, *PCDH7*, *SERPINB9*, *SERPINE2*, *SLC2A3*, *SPN*, *SSC5D*, *TPD52L1*) (Figure 6B, Table S2). Subsequently, we performed GO analysis of these 40 genes and found that the functions were mainly enriched in pathways related to the tumor necrosis factor and co-receptor binding, etc.

Further analysis of the RNA-seq data from the cisplatin group revealed 2140 upregulated genes and 2183 downregulated genes, while the combination group identified 2649 upregulated genes and 2924 downregulated genes, along with 11 genes whose expression was reversed after everolimus treatment (*APOE*, *FOXO1*, *LLGL2*, *MAP2K6*, *N4BP1*, *PDGFB*, *PPAT*, *ZNF114*, *ZNF860* changed from upregulated to downregulated, while *CLEC2D* and *JUND* changed from downregulated to upregulated) (Figure 6E). Figure 6F illustrates the relationships among these 11 unique genes. By cross-referencing with platinum resistance-related databases [31], we identified two specific resistance genes (*FOXO1*, *JUND*) among these eleven genes. We subsequently conducted a PPI (protein–protein interaction) analysis and clustering analysis of these two platinum resistance-related genes alongside the forty unique apoptosis-related genes mentioned earlier. We found that these forty-two genes were divided into five clusters, with the two platinum resistance-related genes grouped together with *TXNIP* in the same cluster. Clusters 1, 2, 3, and 5 are all associated with the cell cycle and cellular development.

## 4. Discussion

Currently, there are no clear treatment consensus or guidelines for SCCOHT internationally, and effectively treating SCCOHT poses a significant challenge [1,2]. In clinical practice, treatment often refers to the guidelines for epithelial ovarian cancer, where patients typically undergo surgery followed by platinum-based combination chemotherapy [2,6]. However, attempts to reuse platinum-based therapies have resulted in chemotherapy failures or severe resistance, leading to recurrence and poor prognosis in SCCOHT patients, which also hinders the application of platinum-based chemotherapy regimens [3,4]. Moreover, significant adverse reactions have affected the conduct of clinical treatments and the use of chemotherapeutic agents. Therefore, there is an urgent need to develop an effective and safe treatment strategy for patients with SCCOHT.

The PI3K/AKT/mTOR pathway is frequently overactivated in various tumors and is associated with the most common mutated genes in tumors and related to the progression, metastasis, and resistance of malignant tumors [32]. In SCCOHT, studies have found that the PI3K/AKT/mTOR pathway is also abnormally activated [13], a conclusion corroborated by our research group’s RNA-seq cohort of SCCOHT patients. Previous studies have demonstrated that inhibiting the PI3K/AKT/mTOR pathway can effectively suppress inflammatory responses and oxidative stress, alleviating tumor progression [32,33,34].

In this study, one of the objectives is to identify drugs that can be used in conjunction with platinum-based therapies to improve or prevent chemotherapy failures and resistance. Based on the findings, we focused on exploring the therapeutic effects of everolimus (an mTOR inhibitor) in SCCOHT. Previous research has shown that everolimus is a derivative of rapamycin (Rapamycin; HY-10219) and an effective, selective, and orally active mTOR1 inhibitor [35,36]. It can effectively interfere with tumor cell growth and proliferation and inhibit tumor angiogenesis, thereby limiting nutrient and oxygen supply and further suppressing tumor growth. It has also been approved for the treatment of various cancers, including renal cell carcinoma, breast cancer, and pancreatic neuroendocrine tumors, demonstrating good therapeutic effects [37,38,39].

The principal findings indicate that everolimus monotherapy significantly inhibits the growth and proliferation of SCCOHT cells while inducing apoptosis [17,34]. Furthermore, the combination of everolimus with cisplatin exerts an additional inhibitory effect on tumor cell proliferation [40]. Flow cytometry analysis reveals a marked increase in apoptosis rates following everolimus treatment, especially in late-stage apoptotic cells, underscoring the role of everolimus in promoting cell death through the activation of apoptotic pathways.

Compared to cisplatin alone, the combination therapy not only significantly enhances cytotoxicity but also induces marked apoptosis and cell cycle arrest. In vivo xenograft model studies confirm that the combined therapy reduces tumor growth without exacerbating common cisplatin-associated side effects, such as weight loss [41,42]. This suggests a favorable therapeutic window for the clinical application of this combination therapy, warranting further investigation into its underlying molecular mechanisms and potential biomarkers of therapeutic response.

The analysis of sequencing results from xenograft models revealed significant molecular phenotype correlations. Specifically, the downregulation of the PI3K/AKT/mTOR signaling pathway (evidenced by decreased levels of p-mTOR and p-AKT) is associated with the observed phenotypic changes, such as reduced cell proliferation and increased apoptosis [43,44,45]. These molecular alterations provide a robust framework for understanding how everolimus enhances the efficacy of cisplatin, potentially offering new therapeutic targets for SCCOHT.

Our findings emphasize the critical role of the PI3K/AKT/mTOR signaling pathway in mediating the response of SCCOHT cells to combined everolimus and cisplatin treatment. The observed downregulation of mTOR and p-mTOR, along with a significant decrease in p-AKT levels post-treatment, highlights the involvement of this pathway in the cytotoxic effects of these drugs [33]. As an mTOR inhibitor, everolimus disrupts the signaling cascades that promote cell proliferation and survival, thereby enhancing the efficacy of cisplatin.

Furthermore, the interaction between mTOR and downstream effectors may elucidate the mechanism by which everolimus enhances cisplatin-induced apoptosis. Multiple research teams have discovered that SCCOHT is characterized by both germline and somatic deleterious mutations [46,47,48,49]. Additionally, *SMARCA4* is the only gene that is recurrently mutated in SCCOHT [47,48,49]. Therefore, pathogenic variants (PVs) of *SMARCA4* are likely the driving mutations in nearly all SCCOHT patients [49]. The *SMARCA4* gene, as part of various *SWI*/*SNF* complexes, is involved in numerous cellular processes, including transcriptional regulation, DNA damage repair, differentiation, and mitosis [1]. Our study demonstrates that everolimus can induce cell cycle arrest, particularly at the G2-M phase [15,50,51]. This suggests that the combination therapy effectively halts cell cycle progression, making SCCOHT cells more susceptible to the cytotoxic effects of cisplatin.

The increase in autophagy markers alongside induced apoptosis highlights the complex interplay between these cellular processes, suggesting that everolimus may enhance cisplatin efficacy through the concurrent regulation of apoptotic and autophagic pathways [14,16,52,53].

In this study, when compared to the DEGs (differentially expressed genes) identified in cisplatin treatment and everolimus treatment, we obtained 40 apoptosis-related genes in the cisplatin + everolimus treatment group. As shown in Figure 6D, there are nine genes that are upregulated (*ARRDC4*, *BMP4*, *C2*, *CTSC*, *DHRS2*, *ISL1*, *TMPRSS5*, *TXNIP*, *XDH*) and thirty-one genes that are downregulated (*COL2A1*, *COLEC11*, *CREB3L1*, *CRLF1*, *CSPG5*, *ENO2*, *ENPP1*, *FGFR2*, *GLDC*, *GRIK2*, *GULP1*, *IFIH1*, *IFIT5*, *IFITM3*, *IRF7*, *KRT8*, *LRP4*, *LRRK2*, *MEGF10*, *MEIS2*, *METTL7A*, *MICA*, *NES*, *OAS1*, *PCDH7*, *SERPINB9*, *SERPINE2*, *SLC2A3*, *SPN*, *SSC5D*, *TPD52L1*). The results of GO analysis of these 40 genes suggest that immune-related pathways such as tumor necrosis factor and co-receptor binding are significantly activated after treatment with everolimus in combination with cisplatin, which suggests that the body’s immune system becomes more active after the combination of the two drugs.

Additionally, after comparing the RNA-seq data from the cisplatin group and the cisplatin + everolimus group, we identified two platinum resistance-related genes, *FOXO1* and *JUND*. Upon performing a protein interaction analysis between these two platinum resistance-related genes and the 40 unique apoptosis-related genes, we found that *TXNIP* is closely related to both *FOXO1* and *JUND*.

Thioredoxin-interacting protein (*TXNIP*), also known as thioredoxin-binding protein 2 (*TBP2*), directly interacts with the major antioxidant protein thioredoxin (*TRX*) and inhibits its antioxidant function and expression [54]. Recent studies have shown that *TRX* is closely associated with cellular stress responses, can induce apoptosis and pyroptosis, and inhibits the proliferation and migration of cancer cells, with its gene expression closely linked to cell cycle processes [54,55]. Research has indicated that overexpressed *TXNIP* can induce cell cycle arrest at the G0/G1 phase [56], a conclusion that aligns with our flow cytometry analysis results and RNA-seq findings. The role of this protein in the synergistic effects of cisplatin and everolimus in SCCOHT still requires further functional analysis and mechanistic exploration.

In summary, we have demonstrated that the combination of cisplatin and everolimus significantly enhances the effects of each drug alone in SCCOHT tumors, indicating a synergistic interaction between the two drugs. We further established that everolimus may regulate tumor cell proliferation by affecting the cell cycle, thereby exerting an inhibitory effect on tumor growth. These findings suggest that the combination of cisplatin and everolimus could be a promising clinical treatment strategy.

## 5. Conclusions

In summary, we have demonstrated that everolimus enhances cisplatin-induced apoptosis and exerts cytotoxic effects on SCCOHT both in vitro and in vivo. Our research also found that everolimus can induce cell cycle arrest, which may partially reverse the adverse effects caused by *SMARCA4* mutations. These studies indicate that the combination of everolimus and cisplatin is a promising clinical treatment approach, and further research is needed to explore the potential effects of related drugs (mTOR inhibitors) in SCCOHT.

## Data Availability

The data generated in this study can be obtained from the corresponding author upon request.

## Supplementary Materials

The following supporting information can be downloaded at https://www.mdpi.com/article/10.3390/biomedicines13010001/s1. Figure S1: The validation of synergistic anti-tumor effect of everolimus and cisplatin in different cell lines by Combenefit software. (A) SCCOHT-CH-1, (B) COV-434; Figure S2: Colony formation of SCCOHT-CH-1 and COV434 with different treatment regimens; Figure S3: EdU incorporation assay with different treatment regimens; Figure S4: (A,B) EdU staining was used to analyze the effects of everolimus and 3-MA on the proliferation of SCCOHT cells (Scale bar = 100 μm). (C,D) Apoptosis measured by Annexin-V and propidum iodide (PI) staining to evaluate the cytotoxic effect of everolimus and 3-MA in SCCOHT cells. Error bars represent the standard deviation, * *p* < 0.05, ** *p* < 0.01, *** *p* < 0.001; Figure S5: Western blot of LC3 and P62 in SCCOHT-CH-1 cells treated with WX390 at different dosages and times; Figure S6: (A) Volcano maps showed differential expressions of autophagy-related genes. (B) Heat maps showing the fold-changes (logarithmic transformation) in autophagy-related genes in different treatment groups. (C) Venn diagram showed the differential expression of autophagy-related genes after different treatments. (D) GOKEGG analysis of the 20 unique autophagy-related genes; Table S1: The data of the tumor volume; Table S2: The list of human apoptosis-related genes.
