# Supplementary figures and images for: mTOR Inhibitor Everolimus Modulates Tumor Growth in Small-Cell Carcinoma of the Ovary, Hypercalcemic Type and Augments the Drug Sensitivity of Cancer Cells to Cisplatin
Source: Biomedicines. 2024 Dec 24;13(1):1. doi: 10.3390/biomedicines13010001 (PMC11759183; doi:10.3390/biomedicines13010001)

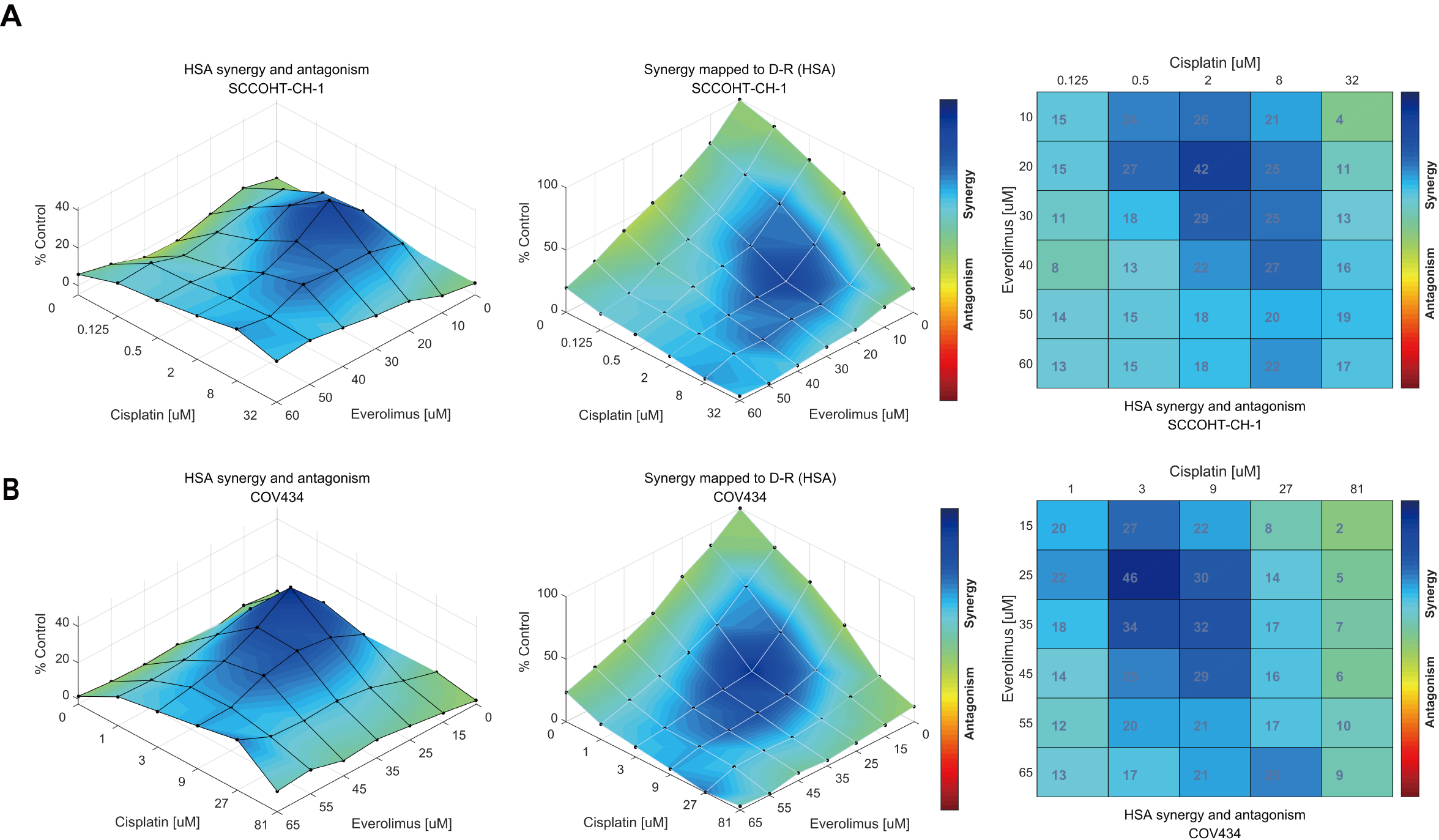

Supplement: Supplementary file 1 [file biomedicines-13-00001-s001.zip › Figure S1.tif]

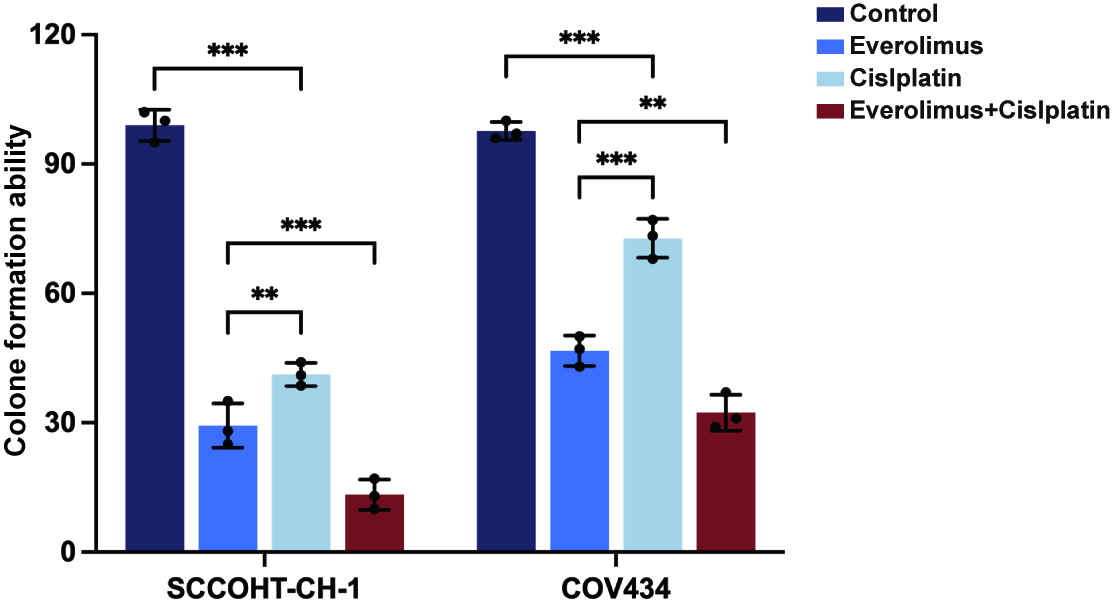

Supplement: Supplementary file 1 [file biomedicines-13-00001-s001.zip › Figure S2.tif]

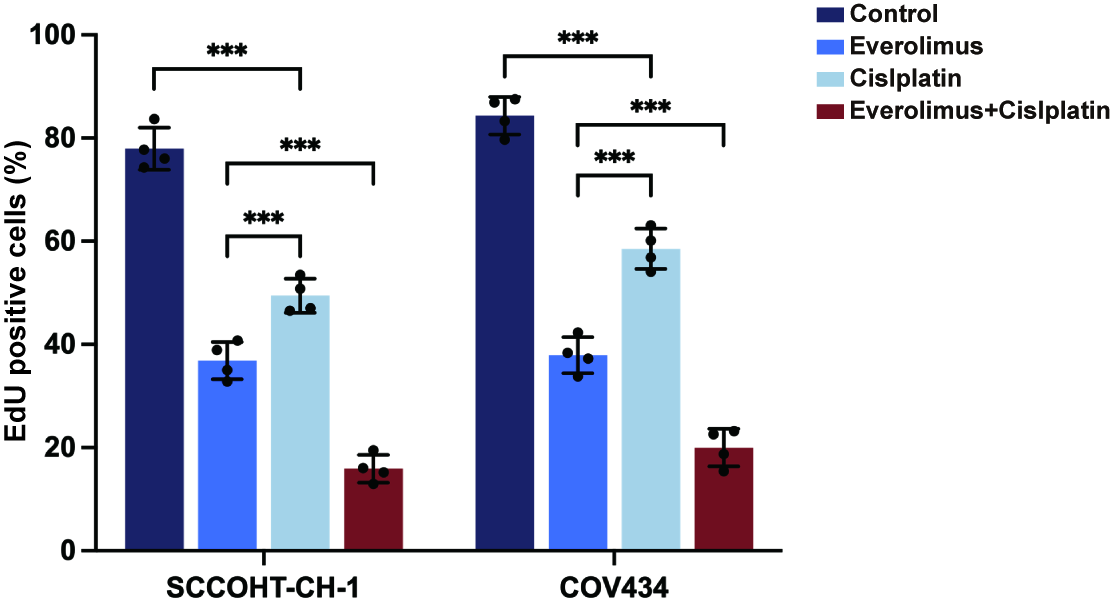

Supplement: Supplementary file 1 [file biomedicines-13-00001-s001.zip › Figure S3.tif]

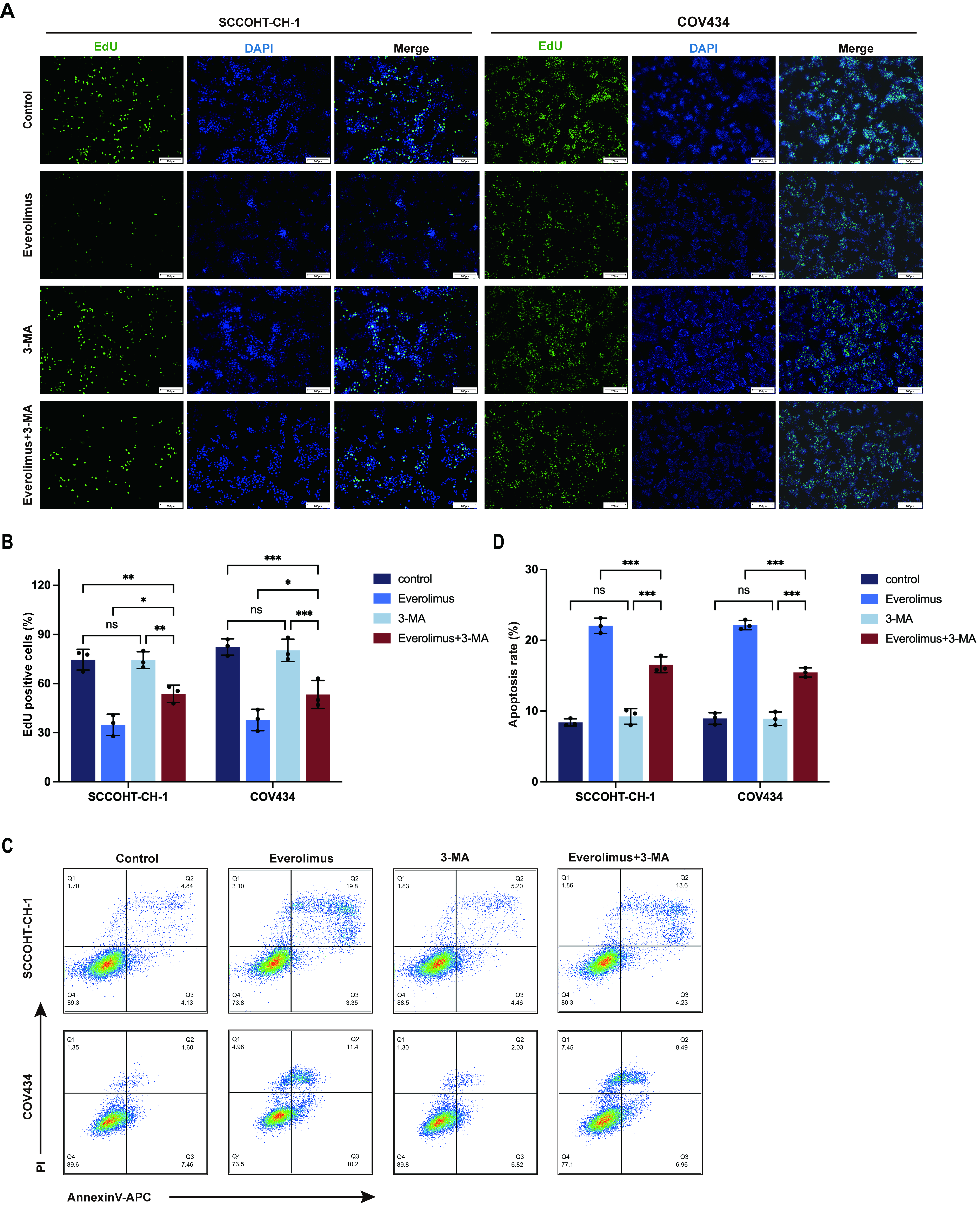

Supplement: Supplementary file 1 [file biomedicines-13-00001-s001.zip › Figure S4.tif]

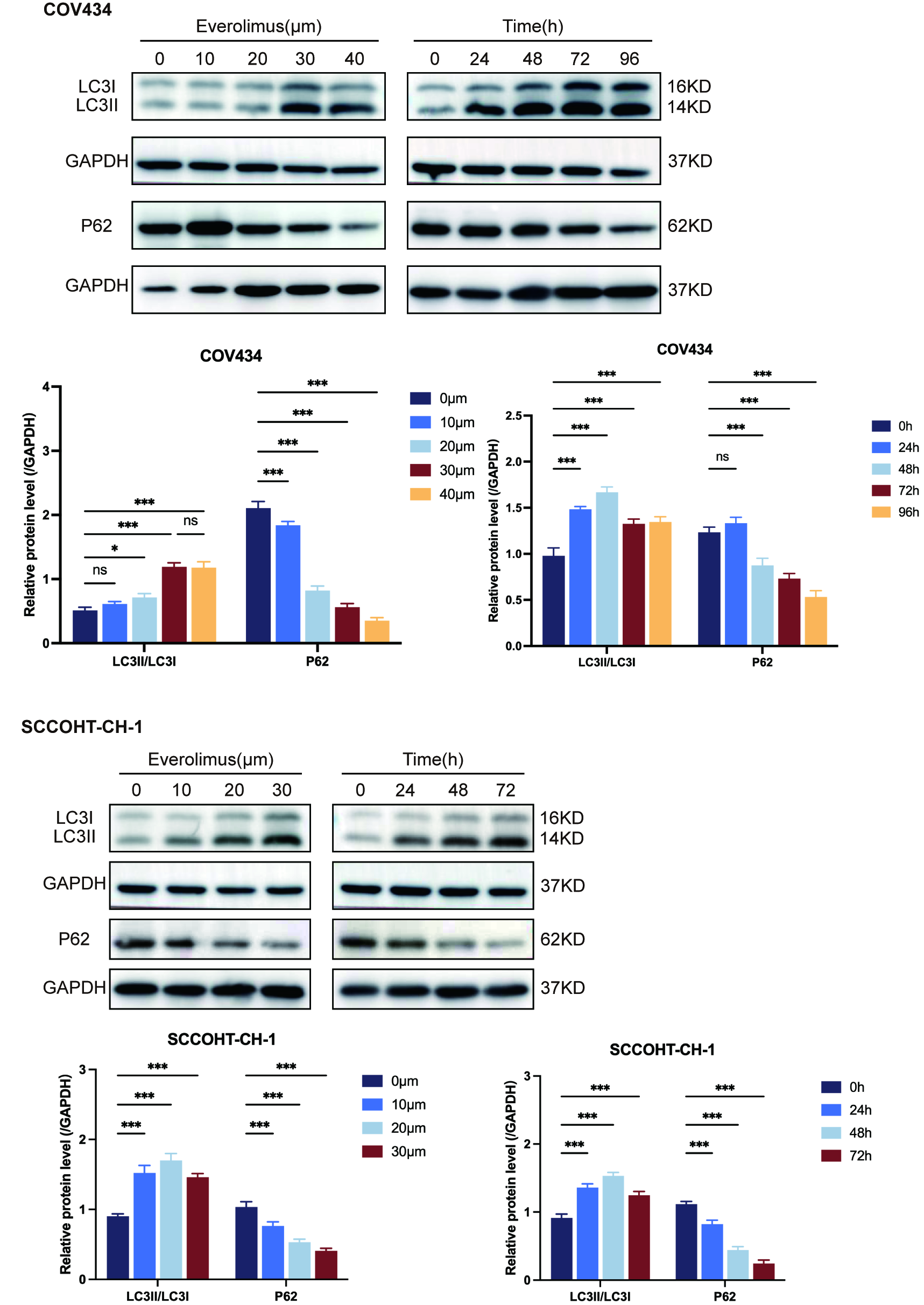

Supplement: Supplementary file 1 [file biomedicines-13-00001-s001.zip › Figure S5.tif]

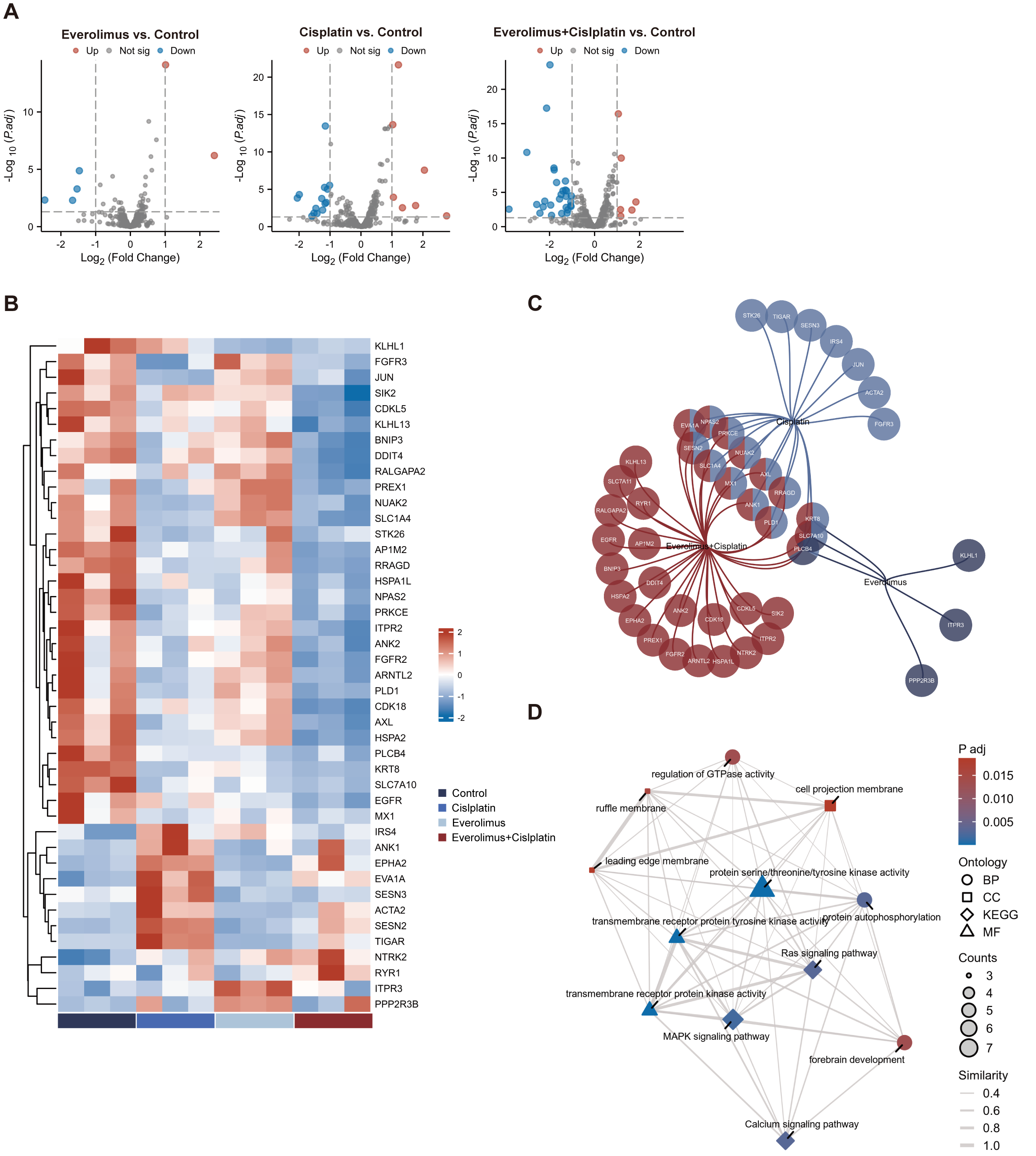

Supplement: Supplementary file 1 [file biomedicines-13-00001-s001.zip › Figure S6.tif]
